# Supplementary material for: Prognostic Implications of Guideline-Directed Medical Therapy for Heart Failure in Functional Mitral Regurgitation: A Systematic Review and Meta-Analysis
Source: Diagnostics (Basel). 2025 Mar 1;15(5):598. doi: 10.3390/diagnostics15050598 (PMC11898837; doi:10.3390/diagnostics15050598)
Supplement: Supplementary file 1 [file diagnostics-15-00598-s001.zip › diagnostics-3469043-supplementary.pdf]

SUPPLEMENTARY MATERIAL

Supplementary Table S1. PRISMA checklist as followed in the current systematic review and meta-analysis.

Supplementary Table S2. Table with excluded studies and rationale.

Supplementary Table S3. Quality assessment of the included studies using the Quality in Prognosis Studies (QUIPS) tool.

Supplementary Table S4. Assessment of the quality of evidence provided by the meta-analysis using the GRADE assessment tool.

Supplementary Figure S1. Funnel plot to assess the risk of bias regarding the association of renin–angiotensin system inhibitors (RASi) with all-cause death in unadjusted models.

Supplementary Figure S2. Funnel plot to assess the risk of bias regarding the association of renin–angiotensin system inhibitors (RASi) with all-cause death in adjusted models.

Supplementary Figure S3. Funnel plot to assess the risk of bias regarding the association of renin–angiotensin system inhibitors (RASi) with composite adverse events in unadjusted models.

Supplementary Figure S4. Funnel plot to assess the risk of bias regarding the association of renin–angiotensin system inhibitors (RASi) with composite adverse events in adjusted models.

Supplementary Figure S5. Funnel plot to assess the risk of bias regarding the association of beta-blockers (BB) with all-cause death in unadjusted models.

Supplementary Figure S6. Funnel plot to assess the risk of bias regarding the association of beta-blockers (BB) with composite adverse events in unadjusted models.

Supplementary Figure S7. Funnel plot to assess the risk of bias regarding the association of beta-blockers (BB) with composite adverse events in adjusted models.

Supplementary Figure S8. Funnel plot to assess the risk of bias regarding the association of mineralocorticoid antagonists (MRA) with all-cause death in unadjusted models.

Supplementary Figure S9. Funnel plot to assess the risk of bias regarding the association of mineralocorticoid antagonists (MRA) with composite adverse events in unadjusted models.

TABLES

Supplementary Table S1. PRISMA checklist as followed in the current systematic review and meta-analysis

**Prognostic implications of guideline-directed medical therapy in functional mitral regurgitation: A meta-analysis**			
**Title**	1	Identify the report as a systematic review.	*Page 1: "Title"*
**ABSTRACT**			
**Abstract**	2	See the PRISMA 2020 for Abstracts checklist.	*Page 2: "Abstract"*
**INTRODUCTION**			
**Rationale**	3	Describe the rationale for the review in the context of existing knowledge.	*Pages 4-5: "Introduction."*
**Objectives**	4	Provide an explicit statement of the objective(s) or question(s) the review addresses.	*Page 6: "Accordingly, the present meta-analysis sought to quantitatively synthesize … for subjects with FMR."*
**METHODS**			
**Eligibility criteria**	5	Specify the inclusion and exclusion criteria for the review and how studies were grouped for the syntheses.	*Page 6: "Eligibility criteria"*
**Information sources**	6	Specify all databases, registers, websites, organisations, reference lists and other sources searched or consulted to identify studies. Specify the date when each source was last searched or consulted.	*Page 5: "Literature Search"*
**Search strategy**	7	Present the full search strategies for all databases, registers and websites, including any filters and limits used.	*Page 5: "Literature Search"*
**Selection process**	8	Specify the methods used to decide whether a study met the inclusion criteria of the review, including how many reviewers screened each record and each report retrieved, whether they worked independently, and if applicable, details of automation tools used in the process.	*Page 6: "Eligibility criteria"*
**Data collection process**	9	Specify the methods used to collect data from reports, including how many reviewers collected data from each report, whether they worked independently, any processes for obtaining or confirming data from study investigators, and if applicable, details of automation tools used in the process.	*Page 7: "Data extraction and data synthesis"*
**Data items**	10a	List and define all outcomes for which data were sought. Specify whether all results that were compatible with each outcome domain in each study were sought (e.g., for all measures, time points, analyses), and if not, the methods used to decide which results to collect.	*Pages 5-7: "Outcomes of interest" + "Data extraction and data synthesis"*
	10b	List and define all other variables for which data were sought (e.g., participant and intervention characteristics, funding sources). Describe any assumptions made about any missing or unclear information.	*Page 7: "Data extraction and data synthesis"*
**Study risk of bias assessment**	11	Specify the methods used to assess risk of bias in the included studies, including details of the tool(s) used, how many reviewers assessed each study and whether they worked independently, and if applicable, details of automation tools used in the process.	*Pages 6-7: "Quality assessment"*
**Effect measures**	12	Specify for each outcome the effect measure(s) (e.g., risk ratio, mean difference) used in the synthesis or presentation	*Pages 7-8: "Data extraction and data synthesis"*

		of results.	
**Synthesis methods**	13a	Describe the processes used to decide which studies were eligible for each synthesis (e.g., tabulating the study intervention characteristics and comparing against the planned groups for each synthesis (item #5)).	*Pages 7-8: "Data extraction and data synthesis"*
	13b	Describe any methods required to prepare the data for presentation or synthesis, such as handling of missing summary statistics, or data conversions.	*Pages 7-8: "Data extraction and data synthesis"*
	13c	Describe any methods used to tabulate or visually display results of individual studies and syntheses.	*Pages 7-8: "Data extraction and data synthesis"*
	13d	Describe any methods used to synthesise results and provide a rationale for the choice(s). If meta-analysis was performed, describe the model(s), method(s) to identify the presence and extent of statistical heterogeneity, and software package(s) used.	*Pages 7-8: "Data extraction and data synthesis"*
	13e	Describe any methods used to explore possible causes of heterogeneity among study results (e.g., subgroup analysis, meta-regression).	*Pages 7-8: "Data extraction and data synthesis"*
	13f	Describe any sensitivity analyses conducted to assess robustness of the synthesised results.	*Pages 7-8: "Data extraction and data synthesis"*
**Reporting bias assessment**	14	Describe any methods used to assess risk of bias due to missing results in a synthesis (arising from reporting biases).	*Pages 7-8: "Data extraction and data synthesis"*
**Certainty assessment**	15	Describe any methods used to assess certainty (or confidence) in the body of evidence for an outcome.	*Pages 7-8: "Data extraction and data synthesis"*
**RESULTS**			
**Study selection**	16a	Describe the results of the search and selection process, from the number of records identified in the search to the number of studies included in the review, ideally using a flow diagram.	*Page 8: "Search Outcomes" + Figure 1*
	16b	Cite studies that might appear to meet the inclusion criteria, but which were excluded, and explain why they were excluded.	*Figure 1+ Table 1*
**Study characteristics**	17	Cite each included study and present its characteristics.	*Pages 8-9: "Study characteristics" + Table 1*
**Risk of bias in studies**	18	Present assessments of risk of bias for each included study.	*Supplementary Table S2 +Page 9: "Study characteristics"*
**Results of individual studies**	19	For all outcomes, present, for each study: (a) summary statistics for each group (where appropriate) and (b) an effect estimate and its precision (e.g., confidence/credible interval), ideally using structured tables or plots.	*Pages 9-10: "Outcome analyses"*
**Results of syntheses**	20a	For each synthesis, briefly summarise the characteristics and risk of bias among contributing studies.	*Pages 9-10: "Outcome analyses"*
	20b	Present results of all statistical syntheses conducted. If meta-analysis was performed, present for each the summary estimate and its precision (e.g., confidence/credible interval) and measures of statistical heterogeneity. If comparing groups, describe the direction of the effect.	*Pages 9-10: "Outcome analyses" +Forest plots*
	20c	Present results of all investigations of possible causes of heterogeneity among study results.	*Pages 9-10: "Outcome analyses" +Forest plots*
	20d	Present results of all sensitivity analyses conducted to assess the robustness of the synthesised results.	*n/a*
**Reporting biases**	21	Present assessments of risk of bias due to missing results	*Pages 10-11: "Quality of*

		(arising from reporting biases) for each synthesis assessed.	*evidence and publication bias assessment"*
**Certainty of evidence**	22	Present assessments of certainty (or confidence) in the body of evidence for each outcome assessed.	*Pages 10-11: "Quality of evidence and publication bias assessment"*
**DISCUSSION**			
**Discussion**	23a	Provide a general interpretation of the results in the context of other evidence.	*Pages 11-15: "Discussion"*
	23b	Discuss any limitations of the evidence included in the review.	*Pages 14-15: "Limitations*
	23c	Discuss any limitations of the review processes used.	*Pages 14-15: "Limitations*
	23d	Discuss implications of the results for practice, policy, and future research.	*Pages 11-15: "Discussion" + Page 15: "Conclusions"*
**OTHER INFORMATION**			
**Registration and protocol**	24a	Provide registration information for the review, including register name and registration number, or state that the review was not registered.	*Page 5: "The current systematic review and meta-analysis was performed in accordance with a prespecified research protocol registered in the PROSPERO database (CRD42024500616)"*
	24b	Indicate where the review protocol can be accessed, or state that a protocol was not prepared.	*Page 5: "The current systematic review and meta-analysis was performed in accordance with a prespecified research protocol registered in the PROSPERO database (CRD42024500616)"*
	24c	Describe and explain any amendments to information provided at registration or in the protocol.	*Not applicable.*
**Support**	25	Describe sources of financial or non-financial support for the review, and the role of the funders or sponsors in the review.	*Page 16: "Sources of funding"*
**Competing interests**	26	Declare any competing interests of review authors.	*Page 16: "Conflict of interest"*
**Availability of data, code and other materials**	27	Report which of the following are publicly available and where they can be found, including: template data collection forms; data extracted from included studies; data used for all analyses; analytic code; any other materials used in the review.	*Page 16: "Data availability"*

Supplementary Table S2. Table with excluded studies and rationale

**Reason for exclusion (n = 31)**	**References of excluded studies**
No outcomes of interest reported	(1-15)
Unable to synthesise reported outcome data	(16)
Other than pre-specified medications	(17-25)
Patients with no FMR are included	(26-29)
Separate outcome data for pre-specified medications not available	(30, 31)
**Reason for exclusion in the sensitivity analysis (n = 5 studies reporting to include patients with multivalvular heart disease)**	**References of excluded studies**
1. Timerniyazi et al., as it included patients with FMR and concomitant aortic stenosis.	(32)
2. Gomez et al. reported to include patients (18% of the total population) with comorbid TR at baseline	(33)
3. Stolfo et al. reported to include patients (41% of the total population) with comorbid TR at baseline	(34)
4. Wu et al. reported to include patients (47% of the total population) with comorbid TR at baseline	(35)
5. Namazi et al. reported to include patients (18% of the total population) with prior tricuspid valvuloplasty at baseline	(36)

Supplementary Table S3. Quality assessment of the included studies using the Quality in Prognosis Studies (QUIPS) tool

Author	Year	Study Participation	Study Attrition	Prognostic Factor Measurement	Outcome Measurement	Study Confounding	Statistical analysis and reporting	Overall assessment
Wu et al.	2005	L	M	L	L	L	L	L
Okura et al.	2016	L	M	M	L	L	L	L
Wada et al.	2016	L	M	L	M	L	L	L
Kim et al.	2017	M	M	M	L	M	L	M
Kubo et al.	2018	L	M	M	L	L	L	L
De Luca et al.	2019	L	L	L	M	M	M	M
Stoflo et al.	2020	M	M	L	M	M	L	M
Namazzi et al.	2020	L	L	L	L	L	L	L
Adamo et al.	2021	L	L	L	L	L	L	L
Tiemuerniyazi et al.	2022	L	M	M	M	L	M	M
Higuchi et al.	2022	M	M	M	M	M	L	M
Gomes et al.	2023	M	M	M	M	L	L	M

The Quality in Prognosis Studies (QUIPS) tool evaluates the individual overall risk of bias of the included studies based on six different domains. Those consist of study participation, study attrition, prognostic factor measurement, outcome measurement, study confounding, statistical analysis, and reporting.

Abbreviations: H, high; L, low; M, moderate

Supplementary Table S4. Assessment of the quality of evidence provided by the meta-analysis using the GRADE assessment tool

Question: Pharmacotherapy and FMR outcomes

Certainty assessment							Certainty		Importance
**Outcome (№ of studies)**	**Study design**	**Risk of bias**	**Inconsistency**	**Indirectness**	**Imprecision**	**Other considerations**	**Hazard Ratio (95% CI)**		
**RASi and all-cause death** (5 studies)	non-randomised studies	Not serious	serious	Not serious	Not serious	Strong association	**HR 0.52** (0.39 to 0.68)	⊕⊕◯◯ Low	IMPORTANT
**Adjusted RASi and all-cause death** (3 studies)	non-randomised studies	Not serious	Very serious	Not serious	serious	none	**HR 0.73** (0.37 to 1.44)	⊕◯◯◯ Very low	IMPORTANT
**RASi and composite adverse events** (6 studies)	non-randomised studies	Not serious	Not serious	Not serious	Not serious	Strong association	**HR 0.54** (0.44 to 0.67)	⊕⊕⊕◯ Moderate	IMPORTANT
**Adjusted RASi and composite adverse events** (7 studies)	non-randomised studies	Not serious	serious	Not serious	Not serious	none	**HR 0.73** (0.56 to 0.95)	⊕◯◯◯ Very low	IMPORTANT
**BB and all-cause death** (5 studies)	non-randomised studies	Not serious	Not serious	Not serious	Not serious	none	**HR 0.62** (0.49 to 0.77)	⊕⊕◯◯ Low	IMPORTANT
**BB and composite adverse events** (9 studies)	non-randomised studies	Not serious	Not serious	Not serious	Not serious	none	**HR 0.62** (0.52 to 0.75)	⊕⊕◯◯ Low	IMPORTANT
**Adjusted BB and composite adverse events** (5 studies)	non-randomised studies	Not serious	serious	Not serious	Not serious	none	**HR 0.60** (0.41 to 0.88)	⊕◯◯◯ Very low	IMPORTANT
**MRA and all-cause death** (3 studies)	non-randomised studies	Not serious	Not serious	Not serious	Not serious	none	**HR 0.88** (0.75 to 1.05)	⊕⊕◯◯ Low	IMPORTANT
**MRA and composite adverse events** (5 studies)	non-randomised studies	Not serious	Not serious	Not serious	Not serious	none	**HR 0.95** (0.81 to 1.12)	⊕⊕◯◯ Low	IMPORTANT

Abbreviations: (a)HR, (adjusted) hazard ratio; BB, beta-blockers; CI, confidence interval; FMR, functional mitral regurgitation; MRA, mineralocorticoid receptor antagonists; RASi, renin–angiotensin system inhibitors

The GRADE tool consists of 8 domains:

i. risk of bias (as assessed through the QUIPS tool),
ii. inconsistency (i.e., heterogeneity in results across studies),
iii. indirectness (i.e., deviation in the research question among the included studies: population characteristics, interventions, or outcome measures),
iv. imprecision (i.e., random variation in outcome estimates due to chance based on sample size, number of events and confidence intervals of the effect estimate),
v. probability of publication bias (as assessed via the visual inspection of the funnel plots),
vi. plausibility of residual confounding (i.e., unmeasured confounding factors that reduce or increase the association of the pharmacotherapy with the outcomes of interest),
vii. dose–response gradient (i.e., existence of a dose–response effect), and
viii. magnitude of effect (i.e., large or very large estimates of the magnitude of the pharmacotherapy effect).

FIGURES

Supplementary Figure S1. Funnel plot to assess the risk of bias regarding the association of renin–angiotensin system inhibitors (RASi) with all-cause death in unadjusted models. Hazard ratio is plotted on the horizontal scale and SE on the vertical axis. The largest and most powerful studies are placed toward the top of the plot.

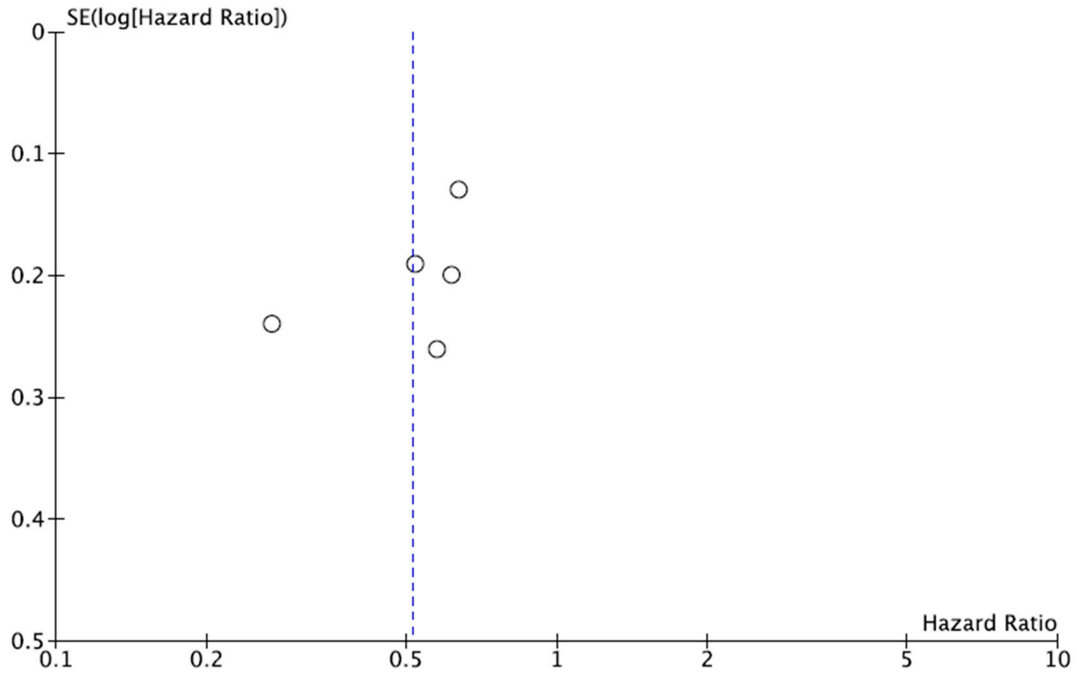

Supplementary Figure S2. Funnel plot to assess the risk of bias regarding the association of renin–angiotensin system inhibitors (RASi) with all-cause death in adjusted models. Hazard ratio is plotted on the horizontal scale and SE on the vertical axis. The largest and most powerful studies are placed toward the top of the plot.

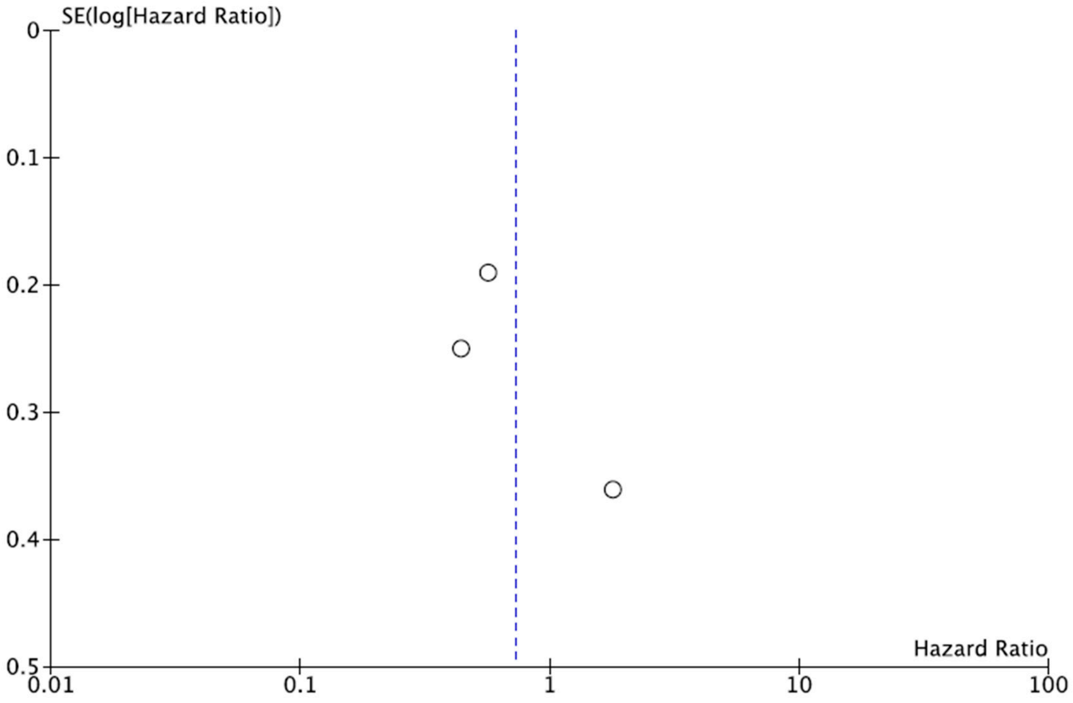

Supplementary Figure S3. Funnel plot to assess the risk of bias regarding the association of renin–angiotensin system inhibitors (RASi) with composite adverse events in unadjusted models. Hazard ratio is plotted on the horizontal scale and SE on the vertical axis. The largest and most powerful studies are placed toward the top of the plot.

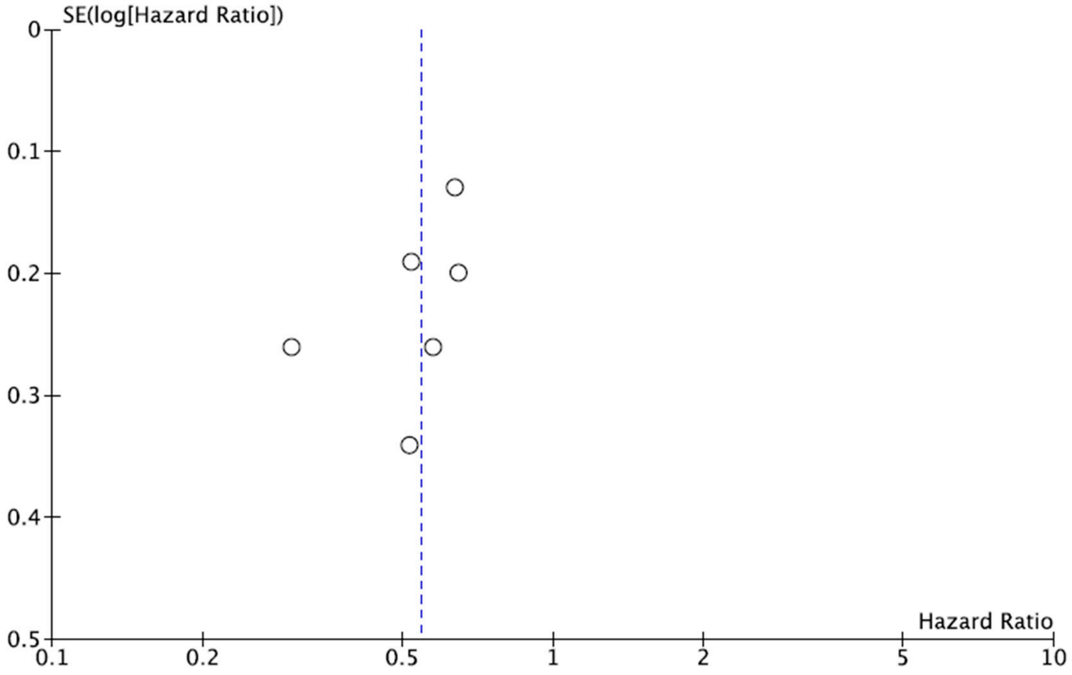

Supplementary Figure S4. Funnel plot to assess the risk of bias regarding the association of renin–angiotensin system inhibitors (RASi) with composite adverse events in adjusted models. Hazard ratio is plotted on the horizontal scale and SE on the vertical axis. The largest and most powerful studies are placed toward the top of the plot.

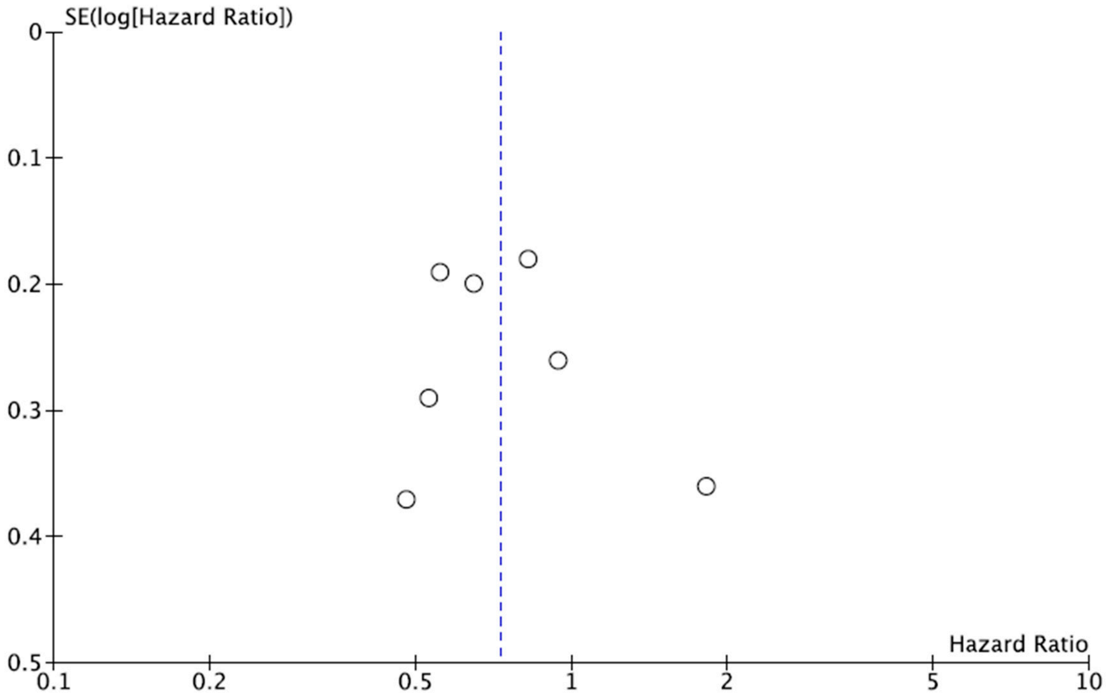

Supplementary Figure S5. Funnel plot to assess the risk of bias regarding the association of beta-blockers (BB) with all-cause death in unadjusted models. Hazard ratio is plotted on the horizontal scale and SE on the vertical axis. The largest and most powerful studies are placed toward the top of the plot.

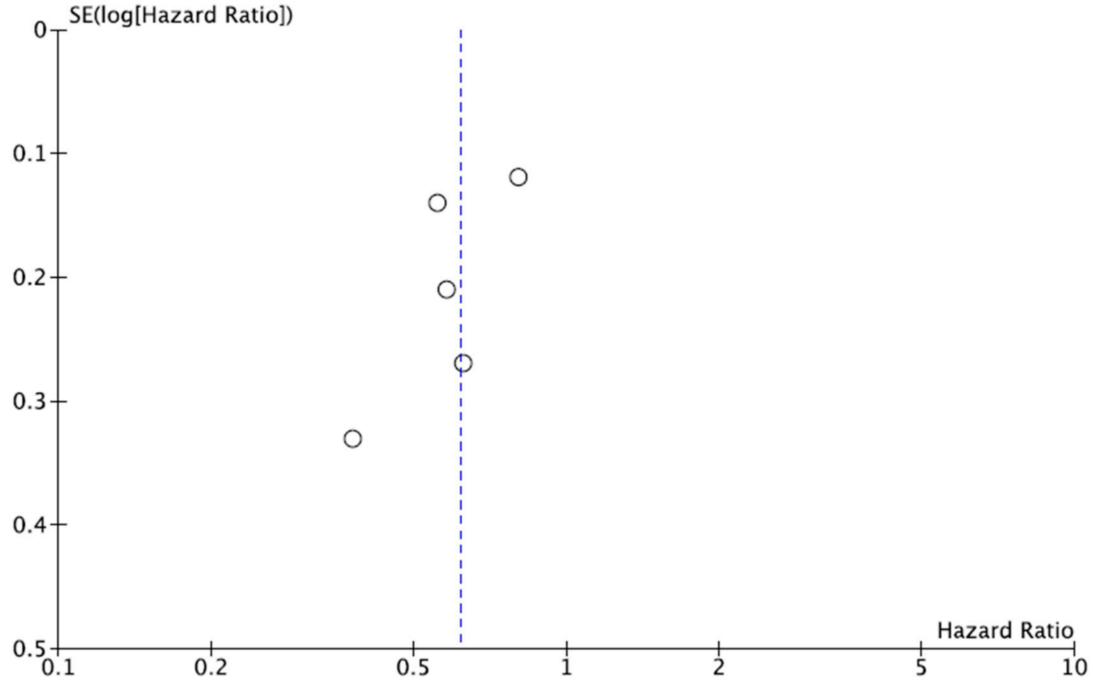

Supplementary Figure S6. Funnel plot to assess the risk of bias regarding the association of beta-blockers (BB) with composite adverse events in unadjusted models. Hazard ratio is plotted on the horizontal scale and SE on the vertical axis. The largest and most powerful studies are placed toward the top of the plot.

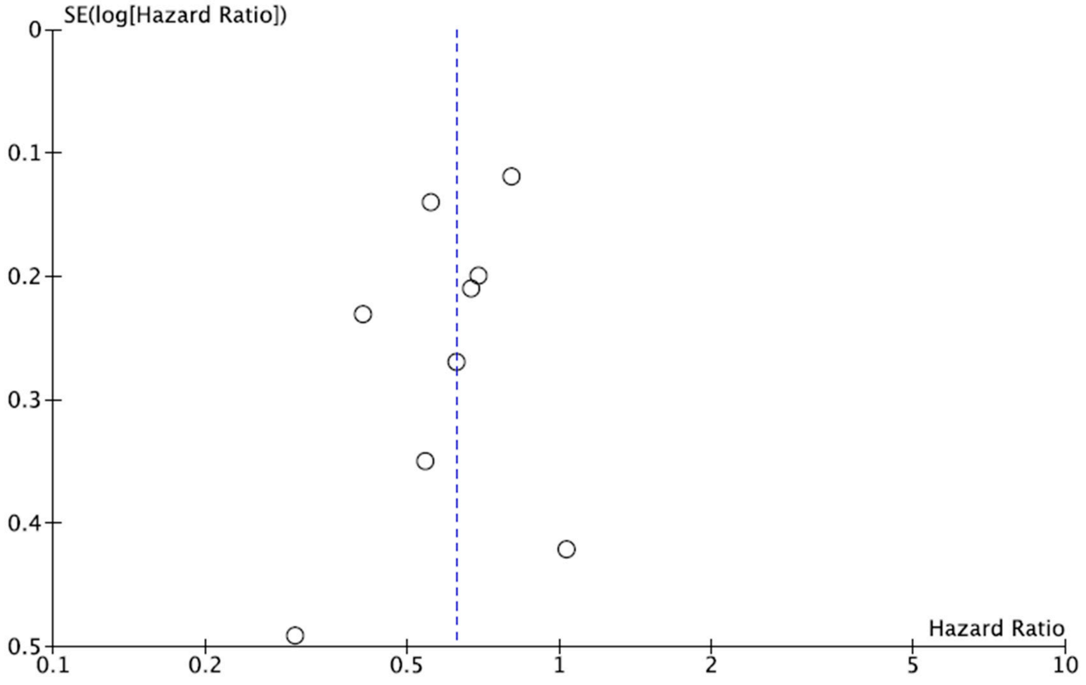

Supplementary Figure S7. Funnel plot to assess the risk of bias regarding the association of beta-blockers (BB) with composite adverse events in adjusted models. Hazard ratio is plotted on the horizontal scale and SE on the vertical axis. The largest and most powerful studies are placed toward the top of the plot.

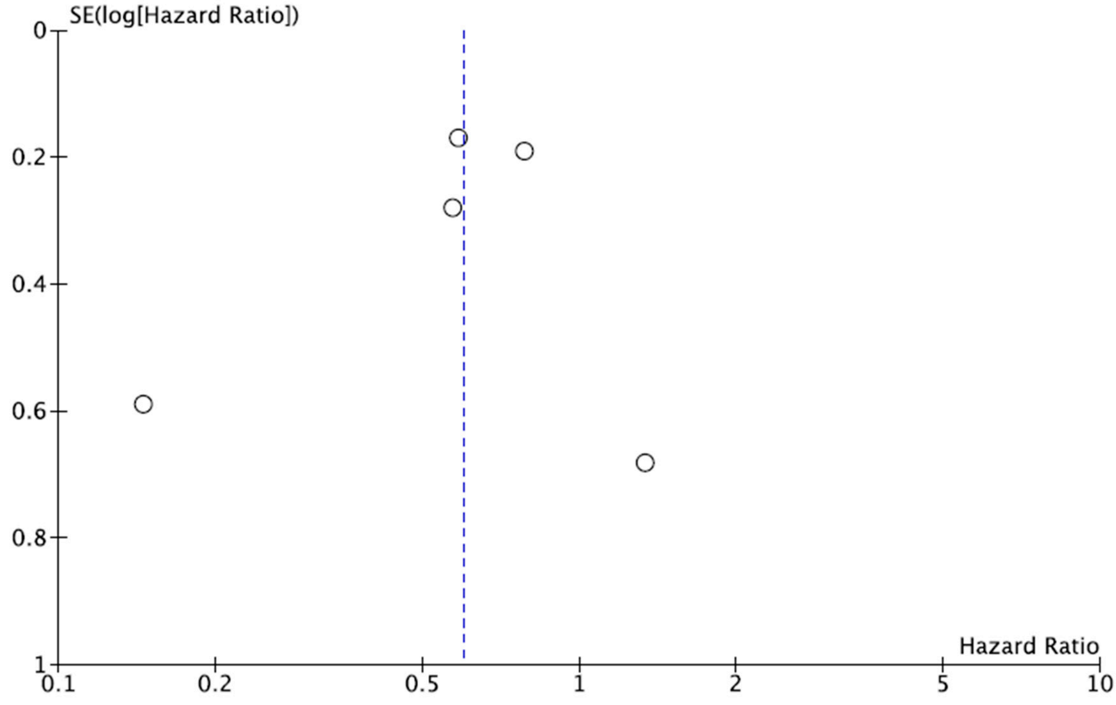

Supplementary Figure S8. Funnel plot to assess the risk of bias regarding the association of mineralocorticoid antagonists (MRA) with all-cause death in unadjusted models. Hazard ratio is plotted on the horizontal scale and SE on the vertical axis. The largest and most powerful studies are placed toward the top of the plot.

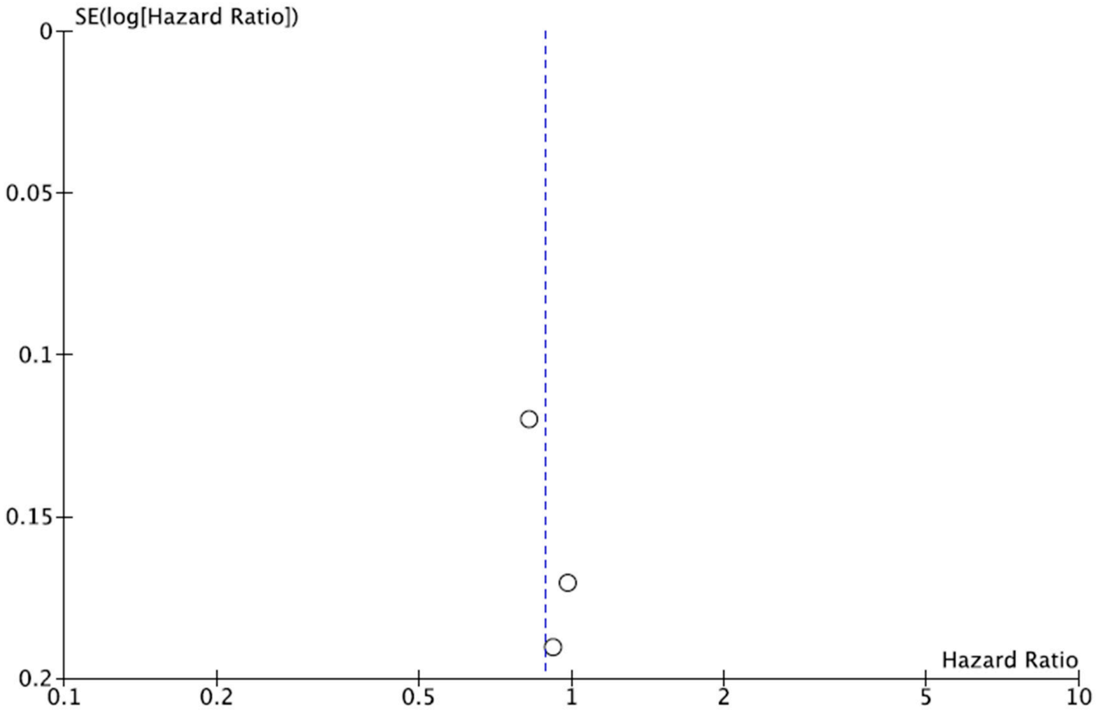

Supplementary Figure S9. Funnel plot to assess the risk of bias regarding the association of mineralocorticoid antagonists (MRA) with composite adverse events in unadjusted models. Hazard ratio is plotted on the horizontal scale and SE on the vertical axis. The largest and most powerful studies are placed toward the top of the plot.

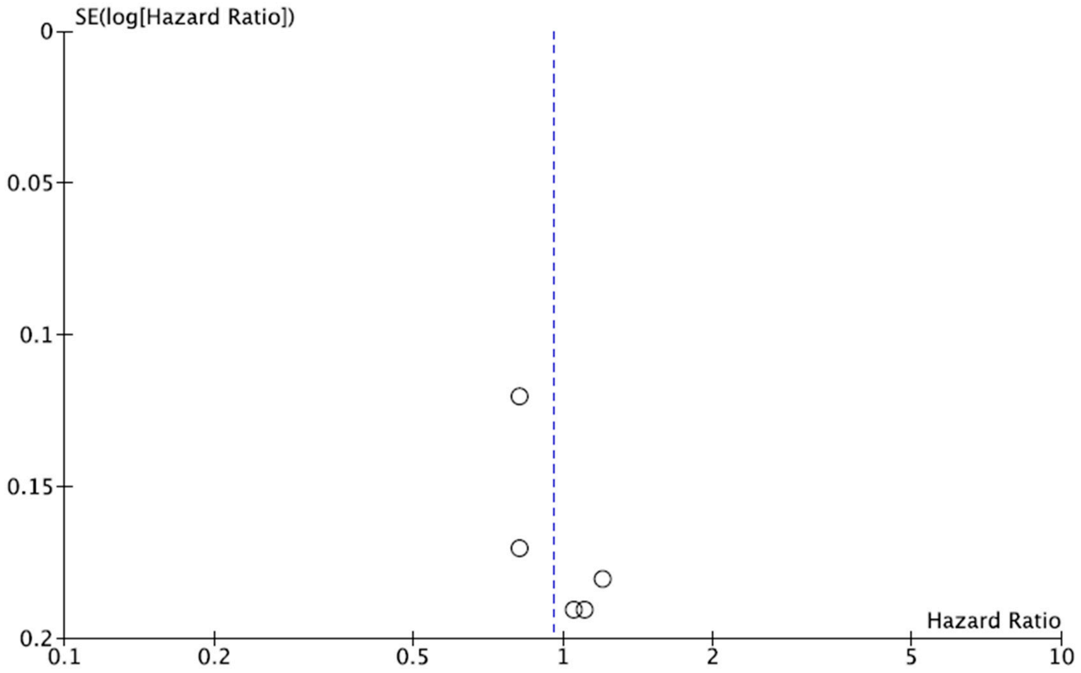

REFERENCES

1. Vijayalakshmi IB, Yavagal ST, Prabhudev N. Role of echocardiography in assessing the mechanism and effect of ramipril on functional mitral regurgitation in dilated cardiomyopathy. Echocardiography. 2005;22(4):289-95.

2. Capomolla S, Febo O, Gnemmi M, Riccardi G, Opasich C, Caporotondi A, et al. Beta-blockade therapy in chronic heart failure: diastolic function and mitral regurgitation improvement by carvedilol. Am Heart J. 2000;139(4):596-608.

3. Comin-Colet J, Sánchez-Corral MA, Manito N, Gómez-Hospital JA, Roca J, Fernández-Nofrerias E, et al. Effect of carvedilol therapy on functional mitral regurgitation, ventricular remodeling, and contractility in patients with heart failure due to left ventricular systolic dysfunction. Transplantation proceedings. 2002;34(1):177-8.

4. Levine AB, Muller C, Levine TB. Effects of high-dose lisinopril-isosorbide dinitrate on severe mitral regurgitation and heart failure remodeling. Am J Cardiol. 1998;82(10):1299-301, a10.

5. Spinka G, Bartko PE, Heitzinger G, Prausmüller S, Winter MP, Arfsten H, et al. Guideline directed medical therapy and reduction of secondary mitral regurgitation. Eur Heart J Cardiovasc Imaging. 2022;23(6):755-64.

6. Waagstein F, Caidahl K, Wallentin I, Bergh CH, Hjalmarson A. Long-term beta-blockade in dilated cardiomyopathy. Effects of short- and long-term metoprolol treatment followed by withdrawal and readministration of metoprolol. Circulation. 1989;80(3):551-63.

7. Kang DH, Park SJ, Shin SH, Hong GR, Lee S, Kim MS, et al. Angiotensin Receptor Neprilysin Inhibitor for Functional Mitral Regurgitation. Circulation. 2019;139(11):1354-65.

8. Lowes BD, Gill EA, Abraham WT, Larrain JR, Robertson AD, Bristow MR, et al. Effects of carvedilol on left ventricular mass, chamber geometry, and mitral regurgitation in chronic heart failure. Am J Cardiol. 1999;83(8):1201-5.

9. Seneviratne B, Moore GA, West PD. Effect of captopril on functional mitral regurgitation in dilated heart failure: a randomised double blind placebo controlled trial. British heart journal. 1994;72(1):63-8.

10. Waagstein F, Strömblad O, Andersson B, Böhm M, Darius M, Delius W, et al. Increased exercise ejection fraction and reversed remodeling after long-term treatment with metoprolol in congestive heart failure: a randomized, stratified, double-blind, placebo-controlled trial in mild to moderate heart failure due to ischemic or idiopathic dilated cardiomyopathy. Eur J Heart Fail. 2003;5(5):679-91.

11. Kotlyar E, Hayward CS, Keogh AM, Feneley M, Macdonald PS. The impact of baseline left ventricular size and mitral regurgitation on reverse left ventricular remodelling in response to carvedilol: size doesn't matter. Heart. 2004;90(7):800-1.

12. Moon MG, Hwang IC, Choi W, Cho GY, Yoon YE, Park JB, et al. Reverse remodelling by sacubitril/valsartan predicts the prognosis in heart failure with reduced ejection fraction. ESC heart failure. 2021;8(3):2058-69.

13. Villani A, Ravaro S, Cerea P, Caravita S, Ciambellotti F, Branzi G, et al. Do the remodeling effects of sacubitril/valsartan treatment depend upon heart failure duration? Journal of cardiovascular medicine (Hagerstown, Md). 2020;21(9):682-7.

14. Adamo M, Pagnesi M, Popolo Rubbio A, Branca L, Grasso C, Denti P, et al. Predictors of optimal procedural result after transcatheter edge-to-edge mitral valve repair in secondary mitral regurgitation. Catheter Cardiovasc Interv. 2022;99(5):1626-35.

15. Kar S, Mack MJ, Lindenfeld J, Abraham WT, Asch FM, Weissman NJ, et al. Relationship Between Residual Mitral Regurgitation and Clinical and Quality-of-Life Outcomes After Transcatheter and Medical Treatments in Heart Failure: COAPT Trial. Circulation. 2021;144(6):426-37.

16. Nogi M, Okura H, Kataoka T, Yoshida K. Predictors and prognostic impact of secondary mitral regurgitation in myocardial infarction with preserved ejection fraction. J Echocardiogr. 2020;18(1):67-72.

17. Aronson D, Mutlak D, Lessick J, Kapeliovich M, Dabbah S, Markiewicz W, et al. Relation of statin therapy to risk of heart failure after acute myocardial infarction. Am J Cardiol. 2008;102(12):1706-10.

18. Nagura F, Kataoka A, Ishibashi R, Mitsui M, Hioki H, Kuwabara M, et al. Effect of oral tolvaptan for 1 year in patients with functional mitral regurgitation. Heart Vessels. 2022;37(3):434-42.

19. Branzi G, Malfatto G, Villani A, Ciambellotti F, Revera M, Giglio A, et al. Acute effects of levosimendan on mitral regurgitation and diastolic function in patients with advanced chronic heart failure. Journal of cardiovascular medicine (Hagerstown, Md). 2010;11(9):662-8.

20. Malfatto G, Della Rosa F, Villani A, Rella V, Branzi G, Facchini M, et al. Intermittent levosimendan infusions in advanced heart failure: favourable effects on left ventricular function, neurohormonal balance, and one-year survival. Journal of cardiovascular pharmacology. 2012;60(5):450-5.

21. Hamilton MA, Stevenson LW, Child JS, Moriguchi JD, Woo M. Acute reduction of atrial overload during vasodilator and diuretic therapy in advanced congestive heart failure. Am J Cardiol. 1990;65(18):1209-12.

22. Keren G, Bier A, Strom JA, Laniado S, Sonnenblick EH, LeJemtel TH. Dynamics of mitral regurgitation during nitroglycerin therapy: a Doppler echocardiographic study. Am Heart J. 1986;112(3):517-25.

23. Keren G, Laniado S, Sonnenblick EH, Lejemtel TH. Dynamics of functional mitral regurgitation during dobutamine therapy in patients with severe congestive heart failure: a Doppler echocardiographic study. Am Heart J. 1989;118(4):748-54.

24. Yedidya I, Lustosa RP, Fortuni F, van der Bijl P, Namazi F, Vo NM, et al. Prognostic Implications of Left Ventricular Myocardial Work Indices in Patients With Secondary Mitral Regurgitation. Circ Cardiovasc Imaging. 2021;14(9):e012142.

25. Verbeke J, Calle S, Kamoen V, De Buyzere M, Timmermans F. Prognostic value of myocardial work and global longitudinal strain in patients with heart failure and functional mitral regurgitation. Int J Cardiovasc Imaging. 2021.

26. Evangelista-Masip A, Bruguera-Cortada J, Serrat-Serradell R, Robles-Castro A, Galve-Basilio E, Alijarde-Guimera M, et al. Influence of mitral regurgitation on the response to captopril therapy for congestive heart failure caused by idiopathic dilated cardiomyopathy. Am J Cardiol. 1992;69(4):373-6.

27. Raphael DM, Liu Z, Jin Z, Cui X, Han D, He W, et al. Effects of sacubitril/valsartan on clinical symptoms, echocardiographic parameters, and outcomes in HFrEF and HFmrEF patients with coronary heart disease and chronic kidney disease. Curr Med Res Opin. 2021;37(7):1071-8.

28. Kataria R, Castagna F, Madan S, Kim P, Saeed O, Adjepong YA, et al. Severity of Functional Mitral Regurgitation on Admission for Acute Decompensated Heart Failure Predicts Long-Term Risk of Rehospitalization and Death. J Am Heart Assoc. 2022;11(1):e022908.

29. Bursi F, Barbieri A, Grigioni F, Reggianini L, Zanasi V, Leuzzi C, et al. Prognostic implications of functional mitral regurgitation according to the severity of the underlying chronic heart failure: a long-term outcome study. Eur J Heart Fail. 2010;12(4):382-8.

30. Tanaka T, Kavsur R, Spieker M, Iliadis C, Metze C, Brachtendorf BM, et al. Guideline-directed medical therapy after transcatheter edge-to-edge mitral valve repair. Heart. 2022;108(21):1722-8.

31. Higuchi S, Orban M, Adamo M, Giannini C, Melica B, Karam N, et al. Guideline-directed medical therapy in patients undergoing transcatheter edge-to-edge repair for secondary mitral regurgitation. Eur J Heart Fail. 2022;24(11):2152-61.

32. Tiemuerniyazi X, Nan Y, Song Y, Yang Z, Zhao W, Xu F, et al. Effect of β-blocker on patients with moderate functional mitral regurgitation undergoing surgical aortic valve replacement. ESC heart failure. 2022;9(5):3317-26.

33. Gomes DA, Lopes PM, Freitas P, Albuquerque F, Reis C, Guerreiro S, et al. Peak left atrial longitudinal strain is associated with all-cause mortality in patients with ventricular functional mitral regurgitation. Cardiovasc Ultrasound. 2023;21(1):9.

34. Stolfo D, Castrichini M, Biagini E, Compagnone M, De Luca A, Caiffa T, et al. Modifications of medical treatment and outcome after percutaneous correction of secondary mitral regurgitation. ESC heart failure. 2020;7(4):1753-63.

35. Wu AH, Aaronson KD, Bolling SF, Pagani FD, Welch K, Koelling TM. Impact of mitral valve annuloplasty on mortality risk in patients with mitral regurgitation and left ventricular systolic dysfunction. J Am Coll Cardiol. 2005;45(3):381-7.

36. Namazi F, van der Bijl P, Hirasawa K, Kamperidis V, van Wijngaarden SE, Mertens B, et al. Prognostic Value of Left Ventricular Global Longitudinal Strain in Patients With Secondary Mitral Regurgitation. J Am Coll Cardiol. 2020;75(7):750-8.
